# Extensively Drug-Resistant *Myroides odoratus* in Critically Ill Patients: A Case Series and Literature Review

**DOI:** 10.1155/2022/6422861

**Published:** 2022-07-14

**Authors:** Melissa O'Neal, Caitlin E. Labay, Jesse E. Harris, William L. Musick, Patricia L. Cernoch, Kevin A. Grimes, Jiejian Lin, Shivan Shah, Elizabeth Ramos-Salazar

**Affiliations:** ^1^Department of Pharmacy, Houston Methodist Hospital, 6565 Fannin St, DB-109, Houston, TX, USA; ^2^Department of Pathology and Genomic Medicine, Houston Methodist Hospital, 6565 Fannin St, Houston, TX, USA; ^3^Divisions of Infectious Disease, Department of Medicine, Houston Methodist Hospital, 6560 Fannin St, Ste 1540, Houston, TX, USA

## Abstract

The bacterial genus *Myroides*, like other members of the *Flavobacteriaceae* family, consists of aerobic, non-motile, Gram-negative bacilli. *Myroides* spp. is considered predominantly opportunistic pathogens as, historically, most documented infections have been in immunocompromised individuals. Along with advancements in molecular assay testing, there are growing reports of clinically relevant *Myroides* spp. infections in immunocompetent individuals. These organisms display broad antimicrobial resistance, and while research into their mechanisms of resistance is progressing, genetic testing has revealed metallo-*β*-lactamases present in their genome. The sporadic identification of *Myroides* spp. and ongoing clarification of resistance patterns make empiric treatment difficult. This report documents two cases of extensively drug-resistant *Myroides odoratus* isolated from critically ill but otherwise immunocompetent patients followed by a review of available literature on *Myroides* spp. antibiotic sensitivities. Our findings indicate that minocycline and moxifloxacin have the highest documented *in vitro* activity against *Myroides* spp.

## 1. Introduction

The bacterial genus *Myroides,* like other members of the *Flavobacteriaceae* family, consists of a group of aerobic, non-motile, Gram-negative bacilli. Differences in motility, fatty acid profiling, and 16S rDNA sequencing led to their reclassification from the *Flavobacterium* genus in 1996 [1]. *Myroides* spp. is considered ubiquitous as their natural reservoir is the aquatic environment, with newer species being identified in seawater; they are not, however, part of typical human microflora. Since their reclassification, eighteen species and two subspecies have been identified with only four, primarily *M. odoratus* and *M. odoratimimus,* being implicated in human infections [2–5]. *Myroides* spp. is considered predominantly opportunistic pathogens as historically most documented infections have been in immunocompromised individuals. Along with advancements in molecular assay testing, there are growing reports of clinically relevant *Myroides* spp. infections in immunocompetent individuals [1, 5, 6]. The described pathogens demonstrate intrinsic broad-spectrum antimicrobial resistance with some isolates displaying the ability to produce biofilms, though the underlying mechanisms of resistance are still being studied [6–8]. The sporadic identification of *Myroides* spp. and ongoing clarification of resistance patterns make empiric treatment difficult. This report documents two cases of extensively drug-resistant *Myroides odoratus* in critically ill but otherwise immunocompetent patients followed by a review of available literature on *Myroides* spp. antibiotic susceptibilities.

## 2. Case 1

The patient is a 48-year-old female with a past medical history comprised of biventricular congestive heart failure status post automatic implantable cardioverter-defibrillator placement, hypertension, hyperlipidemia, and a history of left apical thrombus on warfarin. The patient had no history of underlying immunocompromise or receipt of immunosuppressive therapies. She presented to the emergency department with chest pain, hypotension, nausea, and vomiting. Laboratory results indicated acute renal failure with a lactic acidosis (serum creatinine 1.93 mg/dL, lactic acid 5.5 mmol/L, and potassium 5.3 mEq/L) and elevated B-type natriuretic peptide (BNP 3,470 pg/mL). Transthoracic echocardiogram showed an ejection fraction of <10%, and she was started on inotropes for cardiogenic shock while being worked up for advanced heart failure therapies. While being transferred to the intermediate care unit, she developed pulseless ventricular tachycardia requiring advanced cardiovascular life support and was emergently intubated and transferred instead to the cardiovascular intensive care unit. She was also started on continuous renal replacement therapy (CRRT) at that time due to worsening renal function (serum creatinine 2.05 mg/dL, lactic acid 24.0 mmol/L, and potassium 6.0 mEq/L). The patient began having increased respiratory secretions, chest X-ray was consistent with pulmonary edema, and a bronchoscopy resulted in cultures growing *Klebsiella pneumoniae* which was pan-susceptible except ampicillin. The patient completed 8 days of active therapy with piperacillin-tazobactam transitioned to ceftriaxone and on HD26 a 6-0 cuffed shiley tracheostomy tube was placed. That same day, she was transitioned from CRRT to intermittent hemodialysis. On HD33, she began to have fevers so repeat cultures were drawn and broad-spectrum antibiotics were started empirically. Blood cultures remained negative, but sputum cultures grew multidrug-resistant (MDR) *K. pneumoniae*, prompting a switch to meropenem. After 7 days, she still had leukocytosis (WBC 17.9 × 10^3^ cells with 92% neutrophil predominance) and her procalcitonin was 29.73 ng/mL. A chest CT demonstrated infiltrates which were concerning for atypical pneumonia, prompting the addition of doxycycline. Sputum cultures were recollected, and on HD41 they speciated with pan-resistant *Myroides odoratus* (Table 1). Identification was performed by matrix-assisted laser desorption ionization time-of-flight (MALDI-TOF) mass spectrometry (Bruker Daltonics, MALDI Biotyper® Sirius RUO System, version 4.1). Susceptibility testing and interpretation were achieved by BD Phoenix™ automated identification and susceptibility testing system. Additional susceptibilities were requested but the sample was unrecoverable at that time. Based on improvement in respiratory secretions, doxycycline was continued and ceftazidime/avibactam and aztreonam were added for multidrug resistance while repeat sputum cultures were obtained. MDR *K. pneumoniae* grew, and *M. odoratus* speciated again 3 days later. After 6 days of this therapy, repeat WBC was 11.46 × 10^3^ cells (84.7% neutrophils) and procalcitonin had fallen to 1.24 ng/mL. Repeat *M. odoratus* susceptibilities became available on HD55 (Table 1). Due to the previous clinical worsening before the detection of *M. odoratus* and the repeat isolation of the organism, it was considered a causative pathogen and targeted treatment was employed. The patient was switched from doxycycline to minocycline with nebulized colistin, aztreonam was discontinued, and she remained on ceftazidime/avibactam for MDR *K. pneumoniae*. Nebulized colistin was discontinued after 7 days, and the patient completed 14 days of minocycline for *M. odoratus*. Repeat sputum cultures were negative until HD80, and tracheal aspirate cultures grew *K. pneumoniae* and *M. odoratus*, with similar phenotypes to previous isolates. Only one additional susceptibility was requested for the *M. odoratus* (Table 1), and it was again treated with 14 days of minocycline. The patient completed therapy, repeat sputum cultures were negative, and they were discharged to a long-term acute care facility.

## 3. Case 2

This patient is a 73-year-old male with a past medical history of abdominal aortic aneurysm status post endovascular repair 3 years before presentation, moderate-to-severe aortic stenosis, and coronary artery disease. His hospital course began with a bioprosthetic aortic valve replacement and subsequent double coronary artery bypass. His immediatepostoperative course was complicated by acute hypoxemic respiratory failure, right ventricle failure, and cardiogenic shock requiring inotropes. He was admitted to the cardiovascular ICU and eventually progressed to multisystem organ failure with shock liver, acute kidney injury requiring CRRT, and worsening cardiogenic shock requiring mechanical circulatory support (MCS) with concomitant Impella CP® and ProtekDuo®. He was cardioverted after an episode of monomorphic ventricular tachycardia and found to have a pericardial effusion for which he underwent a pericardial window and thoracostomy. An 8-0 cuffed shiley tracheostomy tube was also placed at that time. He had several infectious complications throughout his hospitalization that included *Escherichia coli* ventilator-associated pneumonia, treated with meropenem and minocycline. A groin wound grew *Pseudomonas aeruginosa* and was treated again with meropenem. However, after 12 days of meropenem, he developed pancytopenia and hematology was consulted to evaluate for potential causes. His WBC at that time was 1.44 × 10^3^ cells, and his absolute neutrophil count was <100 cells. Disseminated intravascular coagulation secondary to an infection, consumption by MCS devices, as well as medications, notably meropenem and colchicine, were included in the differential. He was switched from meropenem to ciprofloxacin to rule out beta-lactam-induced neutropenia, and daily tbo-filgrastim was started. After starting ciprofloxacin, he developed a diffuse macular rash concerning DRESS (53% eosinophilia). All antibiotics were stopped; however, the patient developed worsening shock and repeat blood cultures grew XDR *Citrobacter freundii*. He was started on eravacycline and gentamicin and later switched to eravacycline and amikacin. His central line was replaced, and he was maintained on broad-spectrum antibiotics. On HD62, a CT chest showed multifocal infiltrates and sputum cultures grew MDR *Providencia rettgeri* and MDR *Morganella morganii* prompting the addition of ceftazidime-avibactam. On HD76, serous drainage from a chest wound grew *M. odoratus* (Table 1). Eravacycline was continued for *M. odoratus* coverage, and his remaining antibiotics were escalated to cefiderocol for worsening shock. Two days after sample collection, his Impella CP® was replaced. Repeat blood cultures on HD84 also grew *M. odoratus* and eravacycline was switched to IV minocycline out of concern for nonsusceptibility. While this patient had several previous gram-negative organisms isolated, the isolation of *M. odoratus* alone in blood cultures in addition to clinical worsening led to the decision to consider this the causative pathogen. Notably, this isolate from the blood was phenotypically different from the isolate from his chest wound (Table 1). The patient received 16 days of cefiderocol and minocycline; unfortunately the patient continued to deteriorate and ultimately expired.

## 4. Discussion

We present two cases of *M. odoratus* infection in critically ill patients; one case of ventilator-associated pneumonia caused by *M. odoratus* and *K. pneumoniae*, and one case of *M. odoratus* bacteremia. Aside from critical illness, the patients presented in this report had no known immunocompromise. The patient in Case 2 did develop profound neutropenia during his hospitalization; however, it had fully resolved and colony-stimulating factors had been discontinued more than 2 weeks before the first isolation of *Myroides*. This report is presented to add to the few, but growing, number of cases of pathogenic *Myroides* spp. in immunocompetent individuals described previously by Lu and colleagues [5]. In those cases, an environmental source was identified as a possible route of *Myroides* spp. introduction. Our patients had no known interactions with a contaminated water source or animal bite as described in previous reports [6, 9–12]. The presumed source of infection for these patients was environmental *Myroides* spp. exposure where intubation and procedural wounds, respectively, along with critical illness allowed for bacterial propagation and pathogenesis. *M. odoratus* was ascertained to be a causative pathogen in both cases; in Case 1, the organism speciated after clinical worsening and was correlated to CT findings with repeated detection, whereas in Case 2 the organism was the sole isolate from the first wound and then blood cultures. Furthermore, the identification of *M. odoratus* in these cases was facilitated by MALDI-TOF mass spectrometry, which has demonstrated reliability in differentiating between *Myroides* spp. in concordance with 16s rDNA sequencing [1]. As such, *M. odoratus* while still rare may be considered an emerging pathogen, especially in critically ill or immunocompromised patients.

As seen in our cases, these organisms display broad antimicrobial resistance. Their mechanisms of resistance, while not fully elucidated, are multifaceted, including beta-lactamases, efflux pumps, and altered penetrability via biofilm production [8, 13, 14]. Genomic sequencing has revealed that metallo-*β*-lactamases (MBLs) are intrinsically present within the *M. odoratus* and *M. odoratimimus* genome [13]. Additional analysis has determined the driving beta-lactamases in each species to be TUS-1 and MUS-1, respectively. These beta-lactamases belong to Ambler Class B metalloenzymes responsible for the hydrolysis of a wide range of beta-lactams. Further amino acid comparison showed a similarity of these genes to that of the IND-1 in the closely related *Chryseobacterium indologenes* [13]. Because of the documented presence of these enzymes, the decision was made for the patient in Case 1 to try a combination of ceftazidime/avibactam and aztreonam, which has been evaluated as a therapeutic option in other Gram-negative bacilli that produce MBLs [15]. Once susceptibilities were available, therapy for the *M. odoratus* seen in this case was streamlined to a 14-day course of minocycline. In both cases, minocycline became a mainstay of antibacterial therapy.

Given the relative infrequency of *Myroides* spp. infection and emerging data on its resistance patterns, a PubMed literature search was performed using the terms “*Myroides*” and “infection.” Of the available results, 33 reports including our cases disclosed antimicrobial susceptibility testing (AST) with interpretation (Table 2). The values provided in Table 2 are based on the interpretation reported in each publication representing the assessment guideline (i.e., CLSI or EUCAST) determined most appropriate by each study's investigators. Of note, different methods of susceptibility testing may have been employed between studies. Since the BD Phoenix™ panel used to report susceptibilities in these cases is a broth microdilution test, it was expected to be reliable for reporting minimum inhibitory concentrations (MICs) under the Clinical and Laboratory Standards Institute (CLSI®) M100 Performance Standards for Antimicrobial Susceptibility Testing [16]. The use of automated systems is not expected to drastically alter comparability to manually performed tests; however, if both E-test and microdilution MICs were reported, microdilution results were given deference and used for interpretation. If only the MIC was reported, breakpoints provided by the CLSI® M100 for *Enterobacterales* were used for interpretation when available [16]. FDA breakpoints were used to interpret tigecycline MICs.

To our knowledge, this is one of the largest reviews of *Myroides* spp. susceptibility data and contains a wide range of antibiotics, including results for novel beta-lactam/beta-lactamase inhibitor combinations. Our findings indicate that across the published literature, the agents with the most reliability against *Myroides* spp. are minocycline (100% susceptible) and moxifloxacin (91.8% susceptible). Overall, *Myroides* spp. demonstrated significant resistance to several classes of antibiotics commonly used for Gram-negative infections including polymyxins, cephalosporins, cephamycins, monobactams, and aminoglycosides. Of note, the fluoroquinolones had a minimal activity except for reduced MICs to moxifloxacin, though these results were predominantly driven by one study [17]. Another curiosity seen in the literature, and substantiated by this report, was minocycline susceptibility. All of the isolates found in this search were susceptible to minocycline; however, there was noted nonsusceptibility to tetracycline and only modest susceptibility to tigecycline. This may indicate an alteration in tetracycline resistance gene expression, one of the most studied being tet(X). Tigecycline, a broad spectrum glycylcycline, is purported to evade some of the most common tetracycline resistance genes, notably tet (A–E) but remains vulnerable to tet(X). *In vitro* studies have not only implicated mutations in tet(X) as a mechanism for tigecycline nonsusceptibility but also shown that mutations in another gene, tet(M), can cause tigecycline nonsusceptibility with increased minocycline susceptibility [18]. A recent characterization of tet(X) in clinical isolates included 95 strains of *Flavobacteriaceae* that had confirmed tet(X) presence and displayed tigecycline resistance. The susceptibilities they report are remarkably similar to what is described in Table 2 and notably indicate a high minocycline susceptibility rate of 98.95% [19]. The presence of these genes, while only one piece of the puzzle, offers a hypothesis for the unique resistance patterns seen in this review as well as others.

## 5. Conclusion

We offer this case series and literature review to add to the documentation of clinically relevant isolation of *Myroides* spp. in immunocompetent individuals. Our review indicates that the antibiotics with the most reliability against *Myroides* spp. are minocycline and moxifloxacin. While their mechanisms of resistance are not fully understood, identification of *Myroides* spp. is increasing with the use of advanced molecular testing. These emerging pathogens are increasingly recognized as causing significant disease in both immunocompromised and immunocompetent individuals and as such further genomic characterization is warranted.

## Figures and Tables

**Table 1:** Susceptibilities for *M. odoratus* isolates.

Case 1					
Isolate #1—sputum drug	HD41 MIC (mcg/mL)	Interpretation	HD55 MIC (mcg/mL)	Interpretation	HD80 MIC (mcg/mL)
Amikacin	>32	R	—	—	—
Aztreonam	>16	R	—	—	—
Cefepime	>16	R	—	—	—
Ceftazidime	>16	R	—	—	—
Ceftriaxone	>32	R	—	—	—
Ciprofloxacin	>2	R	—	—	—
Ertapenem	Unavailable	R	—	—	—
Gentamicin	>8	R	—	—	—
Levofloxacin	1	R	—	—	—
Piperacillin/tazobactam	>64/4	R	—	—	—
Tobramycin	>8	R	—	—	—
Trimethoprim/sulfamethoxazole	>2/38	R	—	—	—
Minocycline	—	—	≤1	S	—
Ceftazidime/avibactam	—	—	>8/4	—	—
Ceftolozane/tazobactam	—	—	>8/4	—	—
Meropenem/vaborbactam	—	—	>16/8	—	—
Tigecycline	—	—	—	—	≤1

*Case 2*					
*Isolate #2—wound drug*	*HD76 MIC (mcg/mL)*	*Interpretation*	*Isolate #3—blood HD84 MIC (mcg/mL)*	*Interpretation*	
Amikacin	>32	R	>32	R	
Aztreonam	>16	R	>16	R	
Cefepime	>16	R	>16	R	
Ceftazidime	>16	R	>16	R	
Ceftriaxone	>32	R	>32	R	
Ciprofloxacin	>2	R	>2	R	
Ertapenem	Unavailable	R	Unavailable	R	
Gentamicin	>8	R	>8	R	
Levofloxacin	1	R	>4	R	
Piperacillin/tazobactam	>64/4	R	>64/4	R	
Tobramycin	>8	R	>8	R	
Trimethoprim/sulfamethoxazole	>2/38	R	>2/38	R	
Minocycline	0.064	S	≤1	S	
Meropenem	>32	R	—	—	
Tigecycline	—	—	2	—	
Moxifloxacin	0.094	—	—	—	
Eravacycline	—	—	0.75	—	

HD, hospital day.

**Table 2:** Review of published antimicrobial susceptibility results for *Myroides* spp.

*Myroides* spp.	# Of isolates	#S	% S	Citation
Amikacin	161	1	<1	4–7, 9, 10, 17, 21–34, 36, 40, 41, this report
Amoxicillin or ampicillin	83	28	33.7	13, 17, 28, 32, 35, 36, 41
Amoxicillin/clavulanate	13	4	30.8	4, 10, 13, 28, 32, 36
Aztreonam	156	1	<1	4–6, 10, 13, 17, 21, 22, 25, 26, 28, 30–33, 35, 36, 40, 41, this report
Cefepime	129	1	<1	4–7, 10, 13, 17, 21–23, 25, 26, 28, 30–36, 41, this report
Cefoperazone	43	0	0.0	40, 41
Cefoperazone/sulbactam	14	1	7.1	5, 36, 41
Cefotaxime	13	0	0.0	10, 13, 28, 30, 32, 33
Cefoxitin	10	0	0.0	13, 28, 32
Ceftazidime	159	0	0.0	4–7, 9, 10, 13, 17, 21, 22, 25, 28, 30–36, 38, 40, 41, this report
Ceftazidime/avibactam	2	0	0.0	24, this report
Ceftolozane/tazobactam	1	0	0.0	this report
Ceftriaxone	62	0	0.0	5, 7, 10, 22, 23, 26, 33, 40, 41, this report
Cefuroxime	4	0	0.0	10, 13, 32
Chloramphenicol	21	1	4.8	10, 20, 28, 41
Ciprofloxacin	163	13	8.0	4–7, 9, 10, 17, 20–23, 25–31, 33–36, 38–41, this report
Colistin	91	0	0.0	17, 21, 25, 28, 31, 32, 36, 41
Ertapenem	4	0	0.0	38, this report
Fosfomycin	60	0	0.0	17, 24
Gentamicin	161	0	0.0	4–7, 9, 10, 17, 21–34, 36, 40, 41, this report
Imipenem	158	3	1.9	5, 6, 9, 10, 13, 17, 21–26, 28, 30, 31, 33–36, 38–41
Levofloxacin	102	20	19.6	4, 5, 9, 10, 17, 20, 22, 23, 25, 27, 30, 32, 33, 35, 36, this report
Meropenem	122	32	26.2	4–8, 11, 13, 17, 22, 24–26, 29–34, 36–38, 41, this report
Meropenem/vaborbactam	1	0	0.0	this report
Minocycline	19	19	100.0	31, 41, this report
Moxifloxacin	61	56	91.8	10, 17, this report
Piperacillin/tazobactam	124	17	13.7	4–7, 9, 10, 13, 17, 21–26, 29–33, 35, 36, 41, this report
Tetracycline	41	0	0.0	10, 26, 28, 33, 40
Tigecycline	69	54	78.3	17, 28, 32, this report
Tobramycin	58	1	1.7	4–6, 9, 21, 23, 24, 26–33, 40, this report
Trimethoprim/sulfamethoxazole	155	50	32.3	4–7, 9, 10, 17, 21–24, 28–33, 35, 36, 38, 40, 41, this report

When MICs were available, CLSI breakpoints for *Enterobacterales* were used for susceptibility interpretation. The cases in this report were considered 3 distinct isolates.

## Data Availability

Previously reported data were used to support this study and are cited at relevant places within the text as references [3–15, 17, 20–40].
